# Tamoxifen and Flaxseed Alter Angiogenesis Regulators in Normal Human Breast Tissue In Vivo

**DOI:** 10.1371/journal.pone.0025720

**Published:** 2011-09-30

**Authors:** Ulrika W. Nilsson Åberg, Niina Saarinen, Annelie Abrahamsson, Tarja Nurmi, Sofia Engblom, Charlotta Dabrosin

**Affiliations:** 1 Division of Oncology, Department of Clinical and Experimental Medicine, Faculty of Health Sciences, Linköping University, Linköping, Sweden; 2 Department of Oncology, County Council of Östergötland, Linköping, Sweden; 3 Functional Foods Forum, University of Turku, Turku, Finland; 4 University of Eastern Finland, Kuopio Campus, Kuopio, Finland; Clermont Université, France

## Abstract

The incidence of breast cancer is increasing in the Western world and there is an urgent need for studies of the mechanisms of sex steroids in order to develop novel preventive strategies. Diet modifications may be among the means for breast cancer prevention. Angiogenesis, key in tumor progression, is regulated by the balance between pro- and anti-angiogenic factors, which are controlled in the extracellular space. Sampling of these molecules at their bioactive compartment is therefore needed. The aims of this study were to explore if tamoxifen, one of the most used anti-estrogen treatments for breast cancer affected some of the most important endogenous angiogenesis regulators, vascular endothelial growth factor (VEGF), angiogenin, and endostatin in normal breast tissue in vivo and if a diet supplementation with flaxseed had similar effects as tamoxifen in the breast. Microdialysis was used for in situ sampling of extracellular proteins in normal breast tissue of women before and after six weeks of tamoxifen treatment or before and after addition of 25 g/day of ground flaxseed to the diet or in control women. We show significant correlations between estradiol and levels of VEGF, angiogenin, and endostatin in vivo, which was verified in *ex vivo* breast tissue culture. Moreover, tamoxifen decreased the levels of VEGF and angiogenin in the breast whereas endostatin increased significantly. Flaxseed did not alter VEGF or angiogenin levels but similar to tamoxifen the levels of endostatin increased significantly. We conclude that one of the mechanisms of tamoxifen in normal breast tissue include tipping of the angiogenic balance into an anti-angiogenic state and that flaxseed has limited effects on the pro-angiogenic factors whereas the anti-angiogenic endostatin may be modified by diet. Further studies of diet modifications for breast cancer prevention are warranted.

## Introduction

Long-term exposure to sex steroids such as estradiol and/or progesterone increases the risk of breast cancer [1], [2]. However, the patho-physiological mechanisms behind this increase are still not fully understood. Angiogenesis is a key factor in tumor development and progression [3]. In the female reproductive organs sex steroids are potent regulators of angiogenesis, a process necessary for normal function of these tissues. The breast is also a sex steroid dependent tissue suggesting a potential role for hormonal regulation of angiogenesis of this tissue as well.

One key angiogenic factor, vascular endothelial growth factor (VEGF), has been shown to be estrogen regulated in normal breast tissue [4], [5] and in breast cancer [6], [7], [8]. Another potent pro-angiogenic factor is angiogenin a 14.2 kD polypeptide member of the RNase A superfamily, which induces endothelial cell proliferation after its nuclear translocation [9], [10], [11]. The nuclear translocation of angiogenin is also necessary for the angiogenic response of VEGF, and other angiogenesis regulators [10]. We have recently shown that angiogenin is estrogen regulated in a similar fashion as VEGF in normal breast tissue and experimental breast cancer [4], [5], [12]. However, angiogenesis is regulated by the balance between pro- and anti-angiogenic factors and endostatin is one important endogenous anti-angiogenic protein. Human endostatin is a 20-kDa proteolytic fragment of the C-terminal domain NC1 of collagen XVIII, which inhibits endothelial cell proliferation and migration, and induces apoptosis in proliferating endothelial cells [13], [14], [15]. Endostatin may also down-regulate the VEGF/VEGFR signaling pathway by a direct action on tumor tissue cells, or up-regulate other anti-angiogenic genes [16]. We have previously shown that estradiol down-regulates and tamoxifen up-regulates endostatin generation in experimental breast cancer via effects on matrix metalloproteinase activity [17], [18], [19].

As the incidence of breast cancer is increasing in the Western world there is an urgent need for studies of the mechanisms of sex steroids and anti-estrogens on normal breast tissue in order to develop new preventive strategies for this disease. The anti-estrogen tamoxifen has been shown to reduce the incidence of breast cancer by more than 40%, but this therapy may induce severe side effects such as endometrial cancer and thromboembolism [20], [21], [22]. Diet may be another preventive strategy. In Eastern societies, where the diet contains large amounts of phytoestrogens, the incidence of breast cancer is low and migrant studies support that lifestyle factors rather than genetics affect the risk [23], [24]. One class of phytoestrogen is lignans, which are found in high amounts in flaxseed. In majority of epidemiological studies, dietary plant lignans or their enterolignan metabolites have been associated with reduced breast cancer risk [25].

In the present study we determined if a diet of flaxseed or treatment with tamoxifen affected potent angiogenic regulators in normal human breast tissue *in vivo*.

## Materials and Methods

### Subjects

The regional ethical review board of Linköping approved the study and all women gave their oral informed consents. Written informed consent is not mandatory by the ethical vetting in Sweden. The consent was documented in the research file of each participant. The study was conducted according to the principles expressed in the Declaration of Helsinki.

A total of 36 women were included. Twenty women were pre-menopausal (aged 20–32 years) healthy volunteers with a body mass index (BMI) of median 23.7 range 21–25.4. Of these 20 women, six were investigated in the follicular and luteal phases of one menstrual cycle, five were investigated in two consecutive luteal phases, and nine were investigated in one unexposed luteal phase and one flaxseed exposed luteal phase. Five women were healthy postmenopausal volunteers (aged 52–55 years, BMI median 22.1 range 19.8–24.9) investigated at one time point. Eleven women were postmenopausal (aged 58–78 years, BMI median 23.4 range 21.3–32.7) and were treated for early breast cancer. All patients had ER positive and/or PR positive tumors, ten of the women had tumors sizes between10–19 mm with Elston grade I–II and a negative sentinel node. One woman had a 25 mm Elston grade I tumor with one positive sentinel node and 25 unaffected lymphnodes in the axilla. All women had been off sex steroid containing medication such as contraceptive methods or hormone replacement therapy for more than three months. The premenopausal women had a history of regular menstrual cycles (cycle length 27–34 days) and had not used any antibiotics within past three months.

Biopsies from human breast tissue were obtained from pre-menopausal women, without ongoing hormonal treatment, undergoing routine reduction mammoplasty.

### Experimental design

All women were omnivore without any flaxseed in their daily diet. The premenopausal women were investigated either in the follicular and the luteal phase in one menstrual cycle or during two consecutive luteal phases of two menstrual cycles. The luteinizing hormone (LH) peak was determined in urine samples and thereafter the first microdialysis investigation was performed within 5–10 days. Of the twenty premenopausal women eleven were controls and investigated either in one menstrual cycle or during two consecutive luteal phases without diet modifications whereas nine women added 25 g of freshly ground flaxseed/day to their diet after the first microdialysis session. The next microdialysis investigation was performed at the same day after the LH-peak as in the first un-exposed cycle i.e. if the first microdialysis was performed at day 5 after the LH-peak the next microdialysis investigation was also performed at day 5. The five healthy postmenopausal control women were investigated at one time point.

The exposed postmenopausal women had been treated for early breast cancer and tamoxifen 20 mg/day had been prescribed as an adjuvant therapy. Tamoxifen treatment to early breast cancer patients is a standard regimen in low risk patients in Sweden. All had had a normal breast examination and mammography on the contra-lateral side. Before the start of tamoxifen, microdialysis was performed in the healthy breast and in subcutaneous fat. After six weeks of tamoxifen treatment the women returned for the second microdialysis.

As plasma and serum represent different compartments compared with the extracellular space in tissues, and as they are collected with another technique they are not ideal controls for microdialysates. Therefore, microdialysis performed on abdominal subcutaneous fat was used as an internal control. Additionally, blood samples were collected at the start of each microdialysis investigation.

Prior to insertion of the microdialysis catheters 0.5 ml lidocain (10 mg*/*ml) was administrated intracutaneously. One microdialysis catheter was placed in abdominal subcutaneous fat and one in the upper lateral quadrant of the breast and directed towards the nipple as previously described [4], [26], [27], [28]. At the next microdialysis investigation the catheters were inserted at the same locations as in the previous session. The premenopausal women and the postmenopausal controls were investigated in the left breast whereas the women treated for breast cancer were investigated in their healthy breast. A microdialysis catheter (CMA 71*/*Microdialysis AB, Solna, Sweden), which consists of a tubular dialysis membrane (20 mm long x 0.52 mm in diameter, 100,000 atomic mass cut-off) glued to the end of a double-lumen tube (80 mm long x 0.8 mm in diameter), was used. The catheters were inserted guided by a splitable introducer (CMA Microdialysis AB). The catheters were connected to a microinfusion pump (CMA 107, CMA*/*Microdialysis AB) and perfused with NaCl 154 mmol*/*L and hydroxyethyl starch 60 g/l (Voluven®, Fresenius Kabi, Uppsala, Sweden), at a perfusion rate of 0.5 µl/min. The solution entered the catheter through the outer tube and left it through the inner tube, from which it was collected. After a 30-min equilibration period, the outgoing perfusate containing extracellular proteins was collected and stored at −70°C for subsequent analysis.

Microdialysis is a sampling technique, which allows continuous sampling of the extracellular fluid by passive diffusion of molecules over a semi-permeable membrane. The recovery i.e. the amount of substances from the tissue that diffuse into the perfusion fluid depends on the membrane properties, the flow rate, tissue temperature, and the size of the compound of interest. Diffusion of low molecular substances over the dialysis membrane has been shown to be almost complete at low flow rates using a 30 mm long dialysis membrane [29]. However, for larger molecules the recovery over the membrane decreases and the measured levels in the microdialysis sample cannot be considered as the absolute concentrations in the tissue. The recovery of a certain substance may be measured *in vitro* by putting a microdialysis in a vial containing the compound of interest, perfuse the catheter and divide the concentration of the substance in the dialysate by the concentration in the vial. The *in vitro* recovery of VEGF was 8%, of angiogenin 31%, and of endostatin 33%. This *in vitro* recovery can only be an estimate of the *in vivo* recovery since other factors such as tissue pressure and temperature will affect the diffusion of substances *in vivo*. Therefore, all microdialysis values are given as original raw data without any re-calculations.

### Breast tissue culture

Tissue biopsies containing epithelium, stroma, and adipose tissue, were produced by using an 8 mm biopsy punch (Kai Europe GmbH, Solingen, Germany) and placed in a 12-well plate (Costar, Cambridge, MA, USA). Serum-free medium was used consisting of a 1∶1 mixture of nutrient mixture F-12 (GIBCO, Paisley, UK) and Dulbecco's modified Eagle's medium without phenol red (GIBCO, Paisley, UK) supplemented with transferrin (10 µg/ml; Sigma), insulin (1 µg/ml; Sigma), and bovine serum albumin (0.2 mg/ml; Sigma) with or without physiological levels of 10^−9^ M estradiol (17β-estradiol; E2; Sigma), a combination of 10^−9^ M estradiol and 10^−8^ M progesterone (P4; Sigma) (E2 + P4), or tamoxifen 10^−6^ M. The control group was incubated in media supplemented with the vehicle, ethanol, equivalent to the hormone treated groups (0.001%). The biopsies were treated for 7 days at 37°C in a humidified atmosphere containing 5% CO_2_ and the medium was changed every day. After the seventh day of incubation, the medium from each biopsy was collected and stored at −70°C for subsequent analysis.

### Determinations of estradiol, progesterone, VEGF, angiogenin, and endostatin

Plasma was collected using a plastic tube containing spray dried K2 EDTA as an anticoagulant, and were spun down and frozen at −70°C within 20 minutes of collection.

Microdialysates, plasma samples, and media from the breast tissue biopsies, were analyzed using commercial quantitative immunoassay kits; human endostatin (Quantikine®, R&D Systems, Minneapolis, MN), sensitivity 23 pg/ml and intra-assay variation 3.7–6.9%, VEGF (QuantiGlo® R&D Systems), sensitivity 3.3 pg/ml and intra-assay variation 2.8–7.9%, and Angiogenin Quantikine®, R&D Systems), sensitivity 6 pg/ml and intra-assay variation 2.8–3.3%. Estradiol and progesterone were analyzed using ELISA kits from Calbiotech, Spring Valley, CA. The sensitivity of the estradiol assay was 1.47 pmol/l and the intra-assay variation 4.6–10%, and the sensitivity of the progesterone assay was 0.7 nmol/l and the intra-assay variation 2–5.3%.

### Lignan analyses

Serum samples were prepared for the analyses with a previously described method [30] with slight modifications. Briefly, samples were hydrolyzed at 37°C in 0.2 M sodium acetate buffer pH 5 containing 0.2 U/mL of β-glucuronidase and 2 U/mL of sulphatase and extracted twice with diethyl ether. The extracts evaporated to dryness were re-dissolved in methanol and purified with QAE-Sephadex A-25 in acetate form as earlier described [31]. Chromatographic conditions for serum lignan analyses were as described before [32]. The lignans in the batch of flaxseed used were determined as previously described [33].

### Statistics

Data are expressed as mean±SEM. Student's t-test, ANOVA, and Pearson's correlation coefficient were used as appropriate. A p<0.05 was considered as statistically significant.

## Results

### Characteristics of the subjects

There were no subsequent complications after the microdialysis experiments.

The plasma estradiol levels of the six premenopausal investigated in early follicular phase and luteal phase were 94±20 pmol/L and 322±53 pmol/L respectively, n = 6. The progesterone levels increased from 1.9±0.3 nmol/L to 26±8 nmol/L, n = 6. In the five premenopausal women investigated in two luteal phases the plasma estradiol levels were 334±45 pmol/L in the first luteal phase and 314±61 pmol/l in the second luteal phase, p = 0.8, n = 5, and the progesterone levels were 13±3.9 nmol/l and 11±3 nmol/l respectively, p = 0.5, n = 5. In the flaxseed exposed women the estradiol levels in the unexposed luteal phase were 392±36 pmol/l and in the exposed cycle 425±47 pmol/l, p = 0.6, n = 9. The progesterone levels were 20±3.4 nmol/l and 24±3.8 nmol/L respectively, p = 0.4, n = 9. In the postmenopausal control women the estradiol levels were 64±12 pmol/L and the progesterone levels 1.0±0.1 nmol/L, n = 5. In the postmenopausal women treated for breast cancer the levels before and after tamoxifen treatment were 79±5 pmol/l and 84±4 pmol/l respectively, p = 0.5, n = 11 and the progesterone levels 1.0±0.2 nmol/l and 0.7±0.2 nmol/l respectively, p = 0.4, n = 11. The 25 g of flaxseed contained 44.32 mg of secoisolariciresinol, 0.92 mg of pinoresinol, 0.46 mg of isolariciresinol, 0.40 mg of matairesinol, and 0.24 mg of lariciresinol.

### Correlations

To correlate extracellular levels of VEGF, angiogenin, and endostatin with estradiol, data from unexposed tissues were compared i.e. before start of tamoxifen treatment in the postmenopausal women and in unexposed menstrual cycle in the premenopausal flaxseed group, in the two unexposed luteal phases as well as in the follicular and luteal phases of the premenopausal controls, and in the five healthy postmenopausal women. All premenopausal women and the postmenopausal controls were investigated in both breast tissue and subcutaneous fat whereas of the postmenopausal patients treated for breast cancer, nine were investigated in both in the breast and the fat and two women were investigated in breast tissue only. Hence, 47 microdialysis investigations in unexposed breast tissue and 45 in unexposed fat tissue were performed.

### VEGF

**Figure 1:**
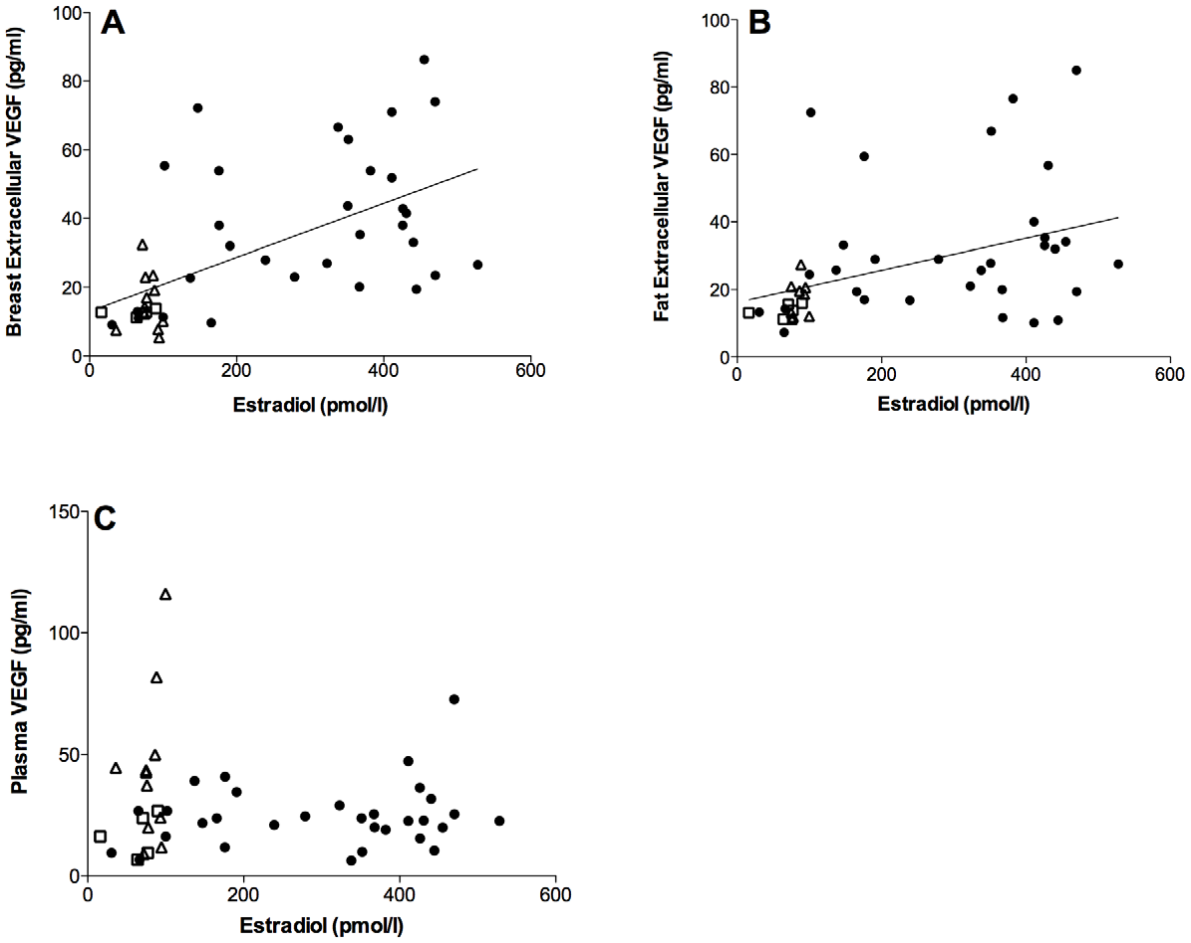
Correlations of VEGF and estradiol. Microdialysis in normal breast tissue and abdominal subcutaneous fat was performed in five sets of women; in follicular and luteal phases of six premenopausal women (12 investigations), in two consecutive luteal phases of five premenopausal women (10 investigations in unexposed tissue), in nine premenopausal women before and after the addition of 25 g of ground flaxseed/day to their diet, in five healthy postmenopausal women, and in 11 women before and after tamoxifen therapy as an adjuvant for early breast cancer (on two women microdialysis was performed in breast tissue only). Estradiol and extracellular and plasma levels of VEGF are presented as raw data from unexposed tissues. Open squares represent healthy postmenopausal women; open triangles represent postmenopausal women with previous breast cancer. A. There were a significant positive correlation between extracellular VEGF in breast tissue and estradiol; r = 0.6, p<0.001, n = 47. B. There were a significant positive correlation between extracellular VEGF in abdominal subcutaneous fat tissue and estradiol; r = 0.4, p<0.01, n = 45. C. There were no correlation between plasma VEGF and estradiol, r = 0.06, p = 0.7, n = 47.

There was a wide inter-individual variation of VEGF levels in all three compartments; breast and fat tissue and plasma. There were a significant positive correlation between extracellular VEGF in breast tissue as well as in fat tissue and estradiol levels; r = 0.6, p<0.001, n = 47 in breast tissue, and r = 0.4, p<0.01, n = 45 in fat tissue, Fig 1A–B. There were no correlation between plasma VEGF and estradiol, r = 0.06, p = 0.7, n = 47, Fig 1C. Plasma progesterone did also correlate significantly with breast VEGF, r = 0.46, p<0.001, n = 47 and fat VEGF, r = 0.35, p<0.01, n = 45. Progesterone did not correlate with extracellular plasma VEGF levels.

### Angiogenin

**Figure 2:**
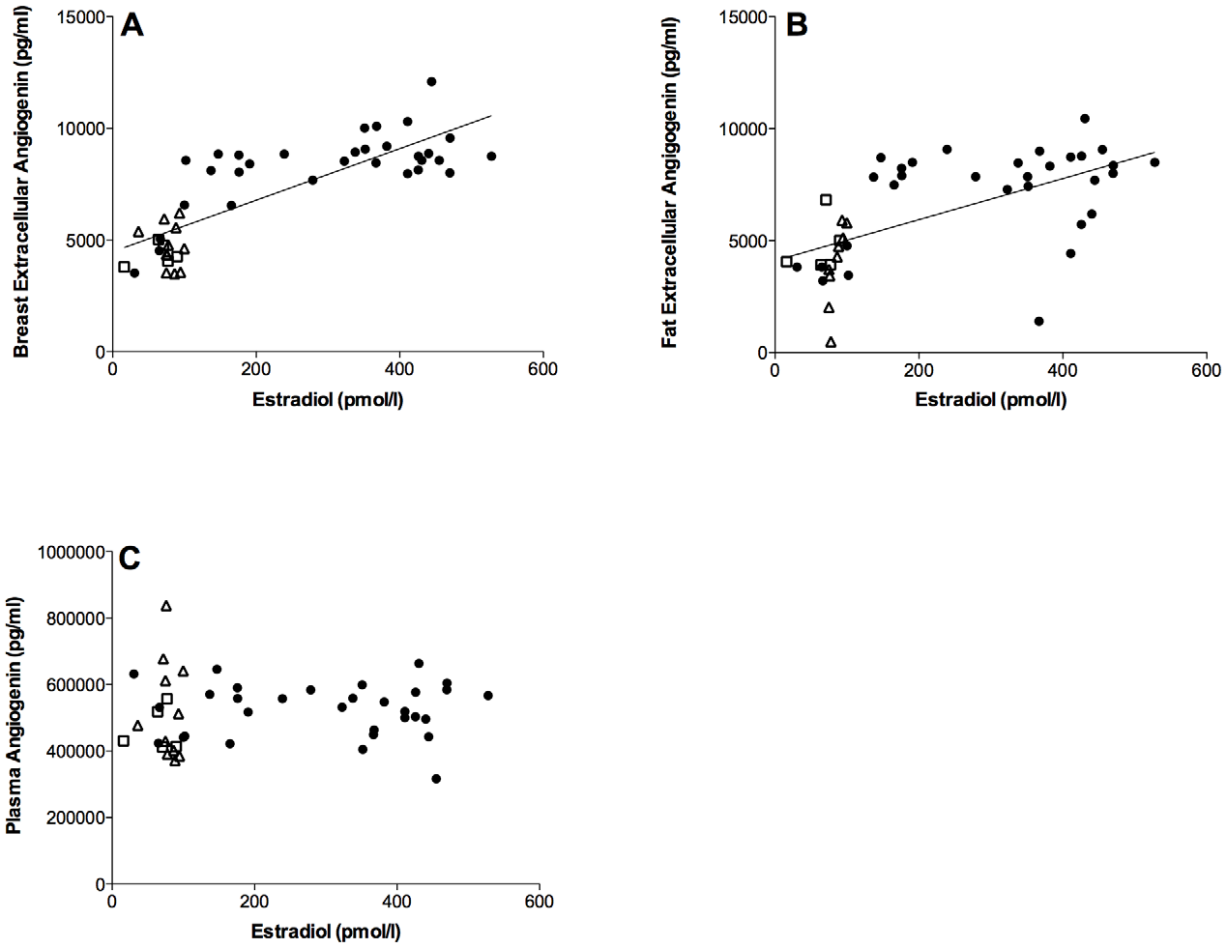
Correlations of angiogenin and estradiol. Microdialysis in normal breast tissue and abdominal subcutaneous fat was performed in five sets of women; in follicular and luteal phases of six premenopausal women (12 investigations), in two consecutive luteal phases of five premenopausal women (10 investigations in unexposed tissue), in nine premenopausal women before and after the addition of 25 g of ground flaxseed/day to their diet, in five healthy postmenopausal women, and in 11 women before and after tamoxifen therapy as an adjuvant for early breast cancer (on two women microdialysis was performed in breast tissue only). Estradiol and extracellular and plasma levels of angiogenin are presented as raw data from unexposed tissues. Open square represents healthy postmenopausal women; open triangle represents postmenopausal women with previous breast cancer. A. There was a strong correlation between estradiol and extracellular breast angiogenin, r = 0.81, p<0.0001, n = 47. B. There was a significant correlation between extracellular fat angiogenin and estradiol, r = 0.6, p<0.0001, n = 45. C. There were no correlation between plasma angiogenin and estradiol, r = 0.06, p = 0.7, n = 47.

There was a strong correlation between estradiol and extracellular breast angiogenin, r = 0.81, p<0.0001, n = 47 and an equally strong significant correlation between extracellular fat angiogenin and estradiol, r = 0.6, p<0.0001, n = 45, Fig 2A–B. There were no correlation between plasma angiogenin and estradiol, r = 0.06, p = 0.7, n = 47, Fig 2C. Plasma progesterone did also correlate significantly with breast angiogenin, r = 0.6, p<0.0001, n = 47 and extracellular fat angiogenin, r = 0.47, p<0.001, n = 45. Progesterone did not correlate with plasma angiogenin levels.

### Endostatin

**Figure 3:**
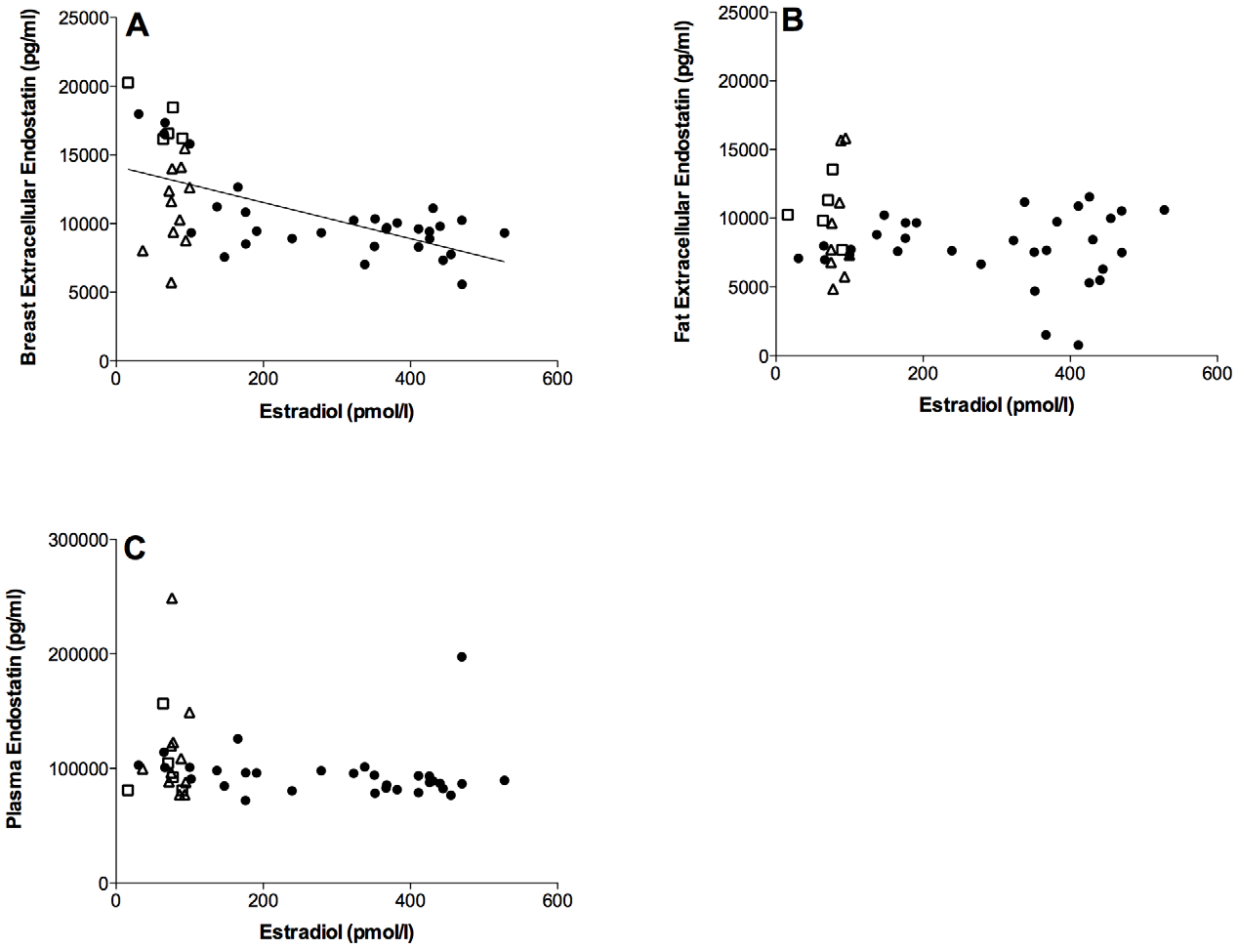
Correlations of endostatin and estradiol. Microdialysis in normal breast tissue and abdominal subcutaneous fat was performed in five sets of women; in follicular and luteal phases of six premenopausal women (12 investigations), in two consecutive luteal phases of five premenopausal women (10 investigations in unexposed tissue), in nine premenopausal women before and after the addition of 25 g of ground flaxseed/day to their diet, in five healthy postmenopausal women, and in 11 women before and after tamoxifen therapy as an adjuvant for early breast cancer (on two women microdialysis was performed in breast tissue only). Estradiol and extracellular and plasma levels of angiogenin are presented as raw data from unexposed tissues. Open square represents healthy postmenopausal women; open triangle represents postmenopausal women with previous breast cancer. A. There was a significant negative correlation between extracellular endostatin levels in unexposed breast tissue and plasma estradiol, r = −0.6, p<0.0001, n = 47. B. Extracellular fat endostatin and estradiol did not show a significant correlation, r = −0.21, p = 0.17, n = 45. C. There was no correlation between plasma endostatin and estradiol, r = −0.19, p = 0.19, n = 47.

There was a significant negative correlation between extracellular endostatin levels in unexposed breast tissue and plasma estradiol, r = −0.6, p<0.0001, n = 47, Fig 3A. Extracellular fat endostatin and estradiol did not show a significant correlation, r = −0.21, p = 0.17, n = 45, Fig 3B. There was no correlation between plasma endostatin and estradiol, r = −0.19, p = 0.19, n = 47, Fig 3C. Plasma progesterone did also correlate significantly with breast endostatin, r = −0.46, p<0.001, n = 47. Progesterone did not correlate with extracellular fat or plasma endostatin levels.

### Hormone exposure to breast tissue biopsies

To explore if sex steroids induced the alterations of the protein levels observed in microdialysates and if tamoxifen had the ability to alter the extracellular levels of these proteins whole breast tissue biopsies from six different premenopausal women were cultured in presence or absence of these compounds.

**Figure 4:**
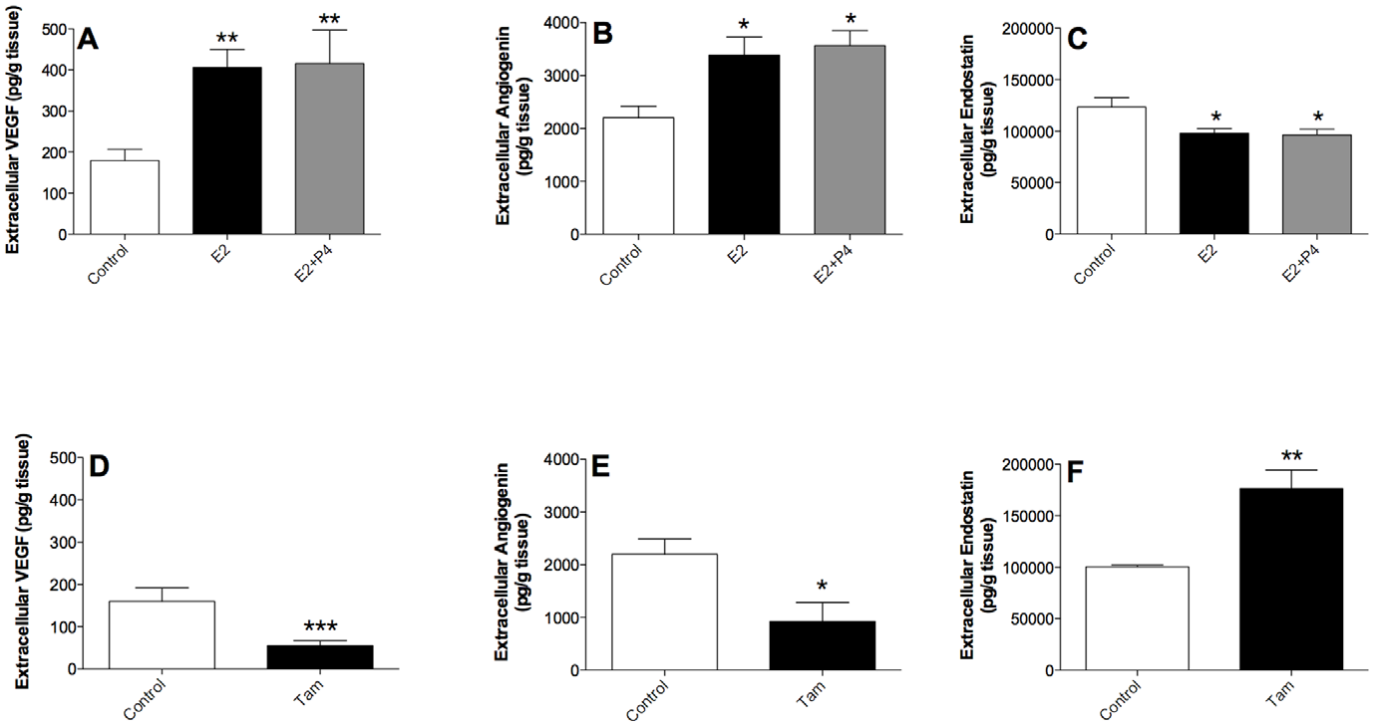
Hormone exposure of breast tissue biopsies ex vivo. Tissue biopsies from pre-menopausal women undergoing reduction mammoplasties were cultured for seven days in serum-free medium supplemented with hormone solvent (C) or in the presence of estradiol (E2; 10^−9^ M), a combination of estradiol and progesterone to mimic the luteal phase of the menstrual cycle (E2+P4; 10^−9^ M and 10^−8^ M, respectively), and tamoxifen (Tam 10^−6^ M). A. Estradiol alone and in combination with progesterone increased the levels of VEGF significantly, **p<0.01, n = 5–6 biopsies in each group. B. Estradiol alone and in combination with progesterone increased the levels of angiogenin significantly, *p<0.05, n = 5–6 biopsies in each group. C. Estradiol alone and in combination with progesterone decreased the levels of endostatin significantly, *p<0.05, n = 5–6 biopsies in each group. D. Tamoxifen decreased the levels of VEGF significantly, ***p<0.001, n = 5–7 biopsies in each group. E. Tamoxifen decreased the levels of angiogenin significantly, *p<0.05, n = 5–7 biopsies in each group. F. Tamoxifen increased the levels of endostatin significantly, **p<0.01, n = 5–7 biopsies in each group.

After exposure to estradiol VEGF and angiogenin levels increased significantly while endostatin levels decreased, Fig 4 A–C. The addition of progesterone did not alter the change compared with estradiol exposure alone, Fig 4A–C.

Tamoxifen decreased the levels of VEGF and angiogenin significantly whereas the levels of endostatin increased by the treatment, Fig. 4D–F.

### In vivo treatments to subjects

Microdialysis was performed in three sets of women; in two consecutive luteal phases of premenopausal women to investigate the reproducibility of the protein measurements in microdialysates; in premeopausal women before and after the addition of flaxseed to their diet. To investigate the compliance in the flaxseed exposed group of women we measured secoisolarisiresinol, enterodiol, and enterolactone levels in serum; secoisolariciresinol increased from non-detectable to 14±8, enterodiol increased from 4.6±1.3 nmol/l to 85±20 nmol/l, and enterolactone increased from 27±7 nmol/l to 189±33 nmol/l confirming compliance with the flaxseed addition. The third cohort of women was patients before and after tamoxifen therapy as an adjuvant for early breast cancer.

### VEGF

**Figure 5:**
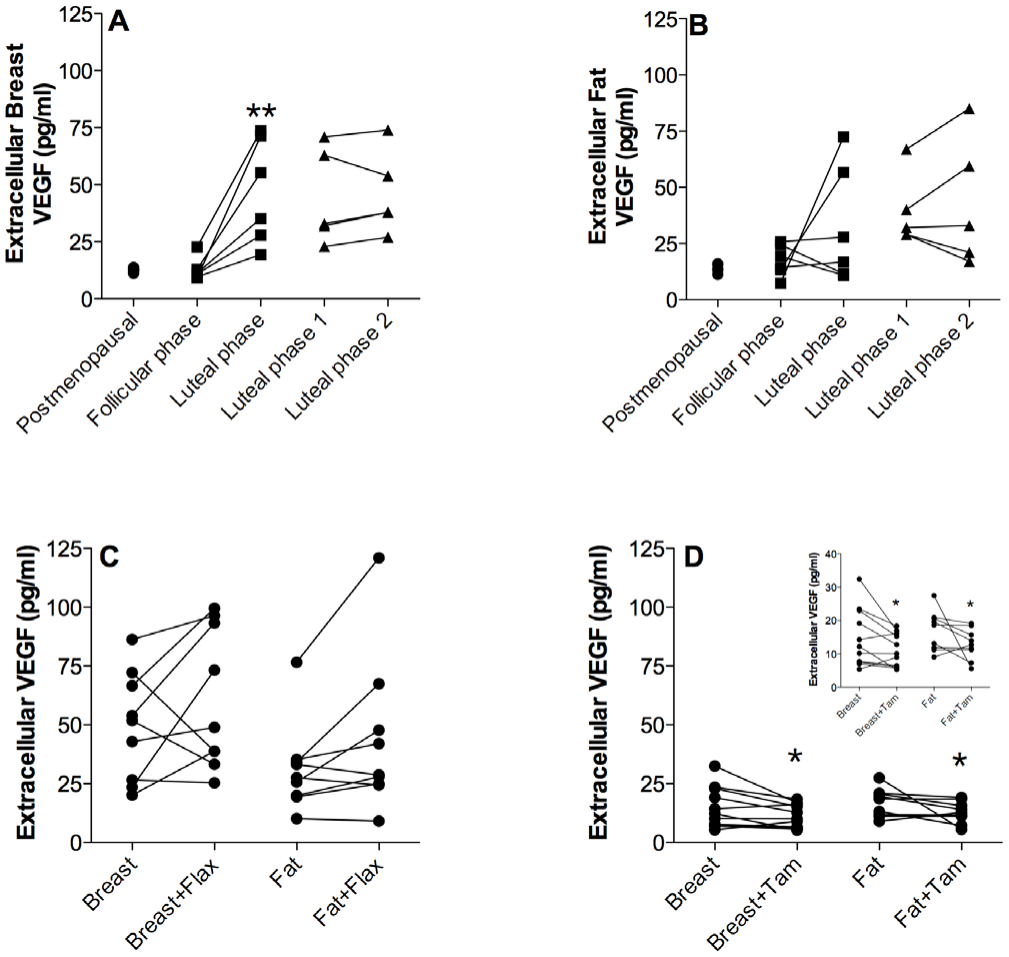
Extracellular VEGF levels in vivo. Microdialysis in normal breast tissue and abdominal subcutaneous fat was performed in five sets of women; in follicular and luteal phases of six premenopausal women, in two consecutive luteal phases of five premenopausal women, in nine premenopausal women before and after the addition of 25 g of ground flaxseed/day to their diet, in five healthy postmenopausal women, and in 11 women before and after tamoxifen therapy as an adjuvant for early breast cancer (on two women microdialysis was performed in breast tissue only). A. There were no changes in extracellular VEGF levels in breast tissue of healthy postmenopausal women and premenopausal women in the follicular phase. VEGF increased significantly in the luteal phase compared to the follicular phase, **p<0.01, n = 6. There were no change during the two consecutive luteal phases, n = 5. B. There were no significant changes in extracellular VEGF in fat tissue. C. There were no significant changes in extracellular breast or fat VEGF levels in the luteal phase after flaxseed supplementation, n = 9. D. Extracellular breast levels of VEGF decreased significantly after six weeks of tamoxifen treatment, *p<0.05, n = 11. As did also the extracellular fat VEGF levels after the treatment period, *p<0.05, n = 9. Inset shows re-scaled tamoxifen data.

In breast tissue, there was a significant increase of VEGF levels in the luteal phase compared with the follicular phase of the menstrual cycle whereas there were no differences in VEGF levels in breast tissue in women investigated in two consecutive luteal phases of the menstrual cycle, Fig 5A. No significant changes were observed in fat tissue, Fig 5B. The addition of flaxseed did not alter the levels significantly either in breast tissue or subcutaneous fat, Fig 5C. Tamoxifen on the other hand decreased the levels significantly both in breast tissue and subcutaneous fat, Fig 5D.

### Angiogenin

**Figure 6:**
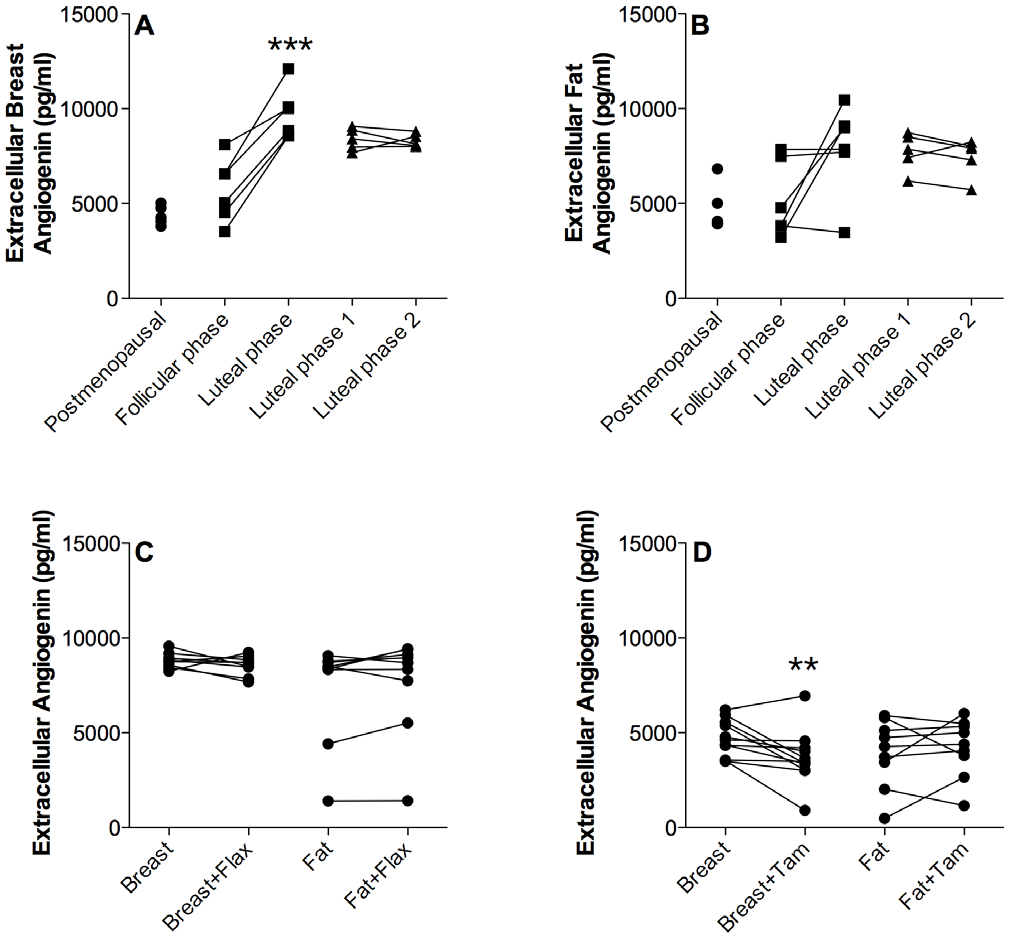
Extracellular angiogenin levels in vivo. Microdialysis in normal breast tissue and abdominal subcutaneous fat was performed in five sets of women; in follicular and luteal phases of six premenopausal women, in two consecutive luteal phases of five premenopausal women, in nine premenopausal women before and after the addition of 25 g of ground flaxseed/day to their diet, in five healthy postmenopausal women, and in 11 women before and after tamoxifen therapy as an adjuvant for early breast cancer (on two women microdialysis was performed in breast tissue only). A. There were no changes in extracellular Angiogenin levels in breast tissue of healthy postmenopausal women and premenopausal women in the follicular phase. Angiogenin increased significantly in the luteal phase compared to the follicular phase, ***p<0.0001, n = 6. There were no change during the two consecutive luteal phases, n = 5. B. There were no significant changes in extracellular Angiogenin in fat tissue. C. There were no significant changes in extracellular breast or fat angiogenin levels in the luteal phase after flaxseed supplementation, n = 9. D. Extracellular breast levels of angiogenin decreased significantly after six weeks of tamoxifen treatment, **p<0.01, n = 11. There were no changes in extracellular fat angiogenin after the treatment period, n = 9.

In breast tissue, there was a significant increase of angiogenin levels in the luteal phase compared with the follicular phase of the menstrual cycle whereas there were no differences in angiogenin levels in breast tissue in women investigated in two consecutive luteal phases of the menstrual cycle, Fig 6A. No significant changes were observed in fat tissue, Fig 6B. The addition of flaxseed did not alter the levels significantly either in breast tissue or subcutaneous fat, Fig 6C. There was a significant decrease of extracellular angiogenin levels of the normal breast after tamoxifen therapy but not in subcutaneous fat, Fig 6D.

### Endostatin

**Figure 7:**
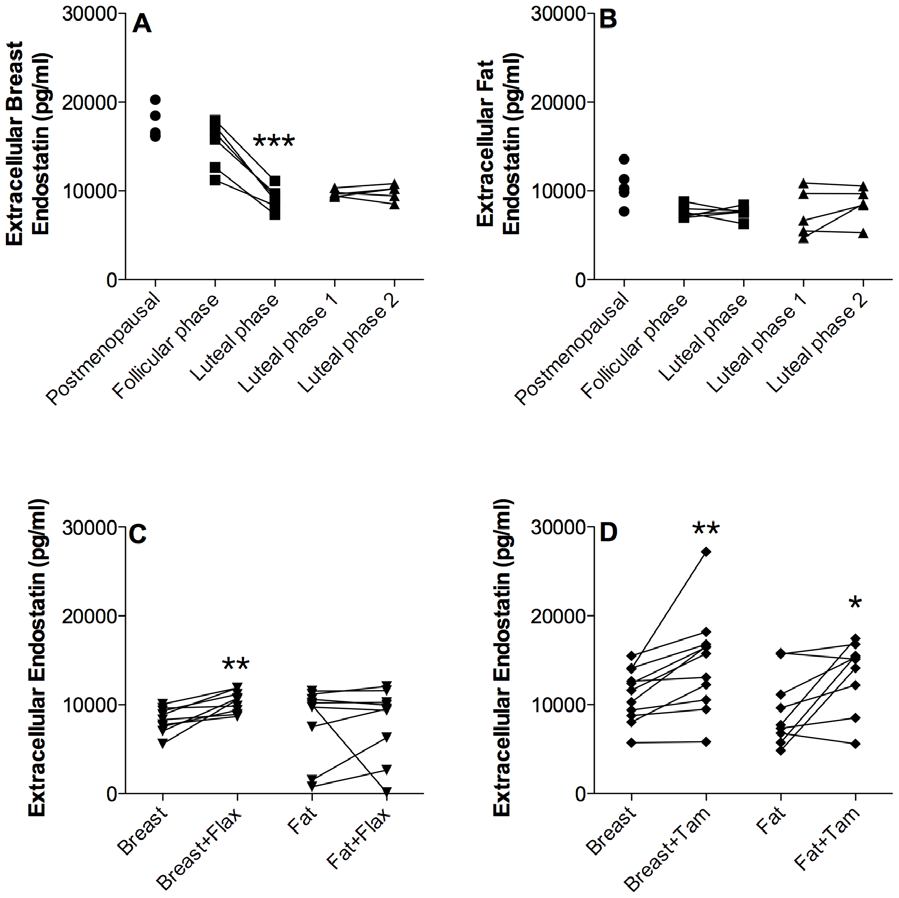
Extracellular endostatin levels in vivo. Microdialysis in normal breast tissue and abdominal subcutaneous fat was performed in five sets of women; in follicular and luteal phases of six premenopausal women, in two consecutive luteal phases of five premenopausal women, in nine premenopausal women before and after the addition of 25 g of ground flaxseed/day to their diet, in five healthy postmenopausal women, and in 11 women before and after tamoxifen therapy as an adjuvant for early breast cancer (on two women microdialysis was performed in breast tissue only). A. There were no changes in extracellular Endostatin levels in breast tissue of healthy postmenopausal women and premenopausal women in the follicular phase. Endostatin decreased significantly in the luteal phase compared to the follicular phase, ***p<0.0001, n = 6. There were no change during the two consecutive luteal phases, n = 5. B. There were no significant changes in extracellular Endostatin in fat tissue. C. There were a significant increase of the extracellular breast endostatin levels in the luteal phase after flaxseed supplementation, **p<0.01, n = 9. The levels of endostatin in fat tissue did not change after a diet of flaxseed. D. Extracellular breast levels of endostatin increased significantly after six weeks of tamoxifen treatment, **p<0.01, n = 11. The levels of endostatin in fat tissue did also increase after the treatment period, *p<0.05 n = 9.

In breast tissue, there was a significant decrease of endostatin levels in the luteal phase compared with the follicular phase, whereas there were no differences in endostatin levels in breast tissue in women investigated in two consecutive luteal phases of the menstrual cycle, Fig 7A. No significant changes were observed in fat tissue, Fig 7B. The addition of flaxseed did increase the levels of endostatin significantly in breast tissue, Fig 7C. However, the tissue endostatin levels did not correlate with serum lignan concentrations e.g. enterodiol, enterolactone, or enterodiol + enterolactone. After tamoxifen the levels of endostatin increased significantly both in breast tissue and subcutaneous fat, Fig 7D.

## Discussion

Here we show that one of the most potent endogenous angiogenesis inhibitor, endostatin, exhibit a negative correlation with estradiol in normal human breast tissue *in situ*. This was confirmed in cultured normal breast tissue biopsies where estradiol exposure decreased the endostatin levels whereas addition of progesterone did not alter endostatin levels compared with estradiol exposure alone. Tamoxifen increased the endostatin levels both in women *in vivo* and in cultured breast biopsies. In a similar fashion as tamoxifen, a diet of flaxseed to healthy premenopausal women increased the extracellular endostatin in breast tissue significantly.

As previously reported from our group [4], [5], [12], two of the most potent endogenous pro-angiogenic factors, extracellular VEGF and angiogenin, exhibited a significant correlation with estradiol in normal breast tissue *in situ* and estradiol exposure to breast tissue biopsies confirmed an estrogen dependent expression of these proteins. Even though the *in vivo* levels of the proteins in breast tissue exhibited significant correlations with progesterone levels the breast biopsy experiment showed that progesterone had no additional effect compared to estradiol exposure alone. Tamoxifen significantly decreased the levels of VEGF and angiogenin in normal breast tissue both in microdialysates of women and in media of cultured breast tissue biopsies. A diet of flaxseed did, however, not alter VEGF or angiogenin in the breast.

Estrogens have been shown to regulate angiogenesis in experimental breast cancer, but very few studies have investigated the regulation of angiogenesis in the normal human breast [5], [7], [8]. One reason has been a lack of suitable techniques and models for this research. Microdialysis is a minimally invasive technique, which enables sampling of molecules in the extracellular space *in situ* of normal breast and whole breast tissue culture enables studies of the biology of the breast as an organ *ex vivo* [18], [34], [35], [36]. The extracellular space is the bioactive site for the majority of angiogenic factors as a result of the release and/or cleavage of larger proteins into anti-angiogenic fragments. For example, endostatin is cleaved in the extracellular space from collagen XVIII. Due to this post-translational activation/release mRNA levels or intracellular protein levels will not reflect bioactive protein levels as we previously have shown regarding the VEGF regulation [37]. Moreover, as in the case of endostatin, antibodies raised against cleaved proteins may cross-react with the primary molecule, in the case of endostatin, collagen XVIII. By using microdialysis sampling of proteins from their bioactive site with a membrane cut-off level that excludes larger original proteins only the cleaved bioactive fragment is sampled. One of the strengths in this study is that a direct measurement of the proteins in live tissue is used and in a unique manner quantifies proteins direct in the target organ.

A tipping of the angiogenic balance to favor a pro-angiogenic environment is a key event in the initiation, growth, and progression of tumors. At autopsy it has been found that up to fifteen percent of all women present *in situ* tumors in their breast whereas only 1% of women in the same age range are diagnosed with breast cancer [38]. An angiogenic switch may convert this reservoir of non-clinical detectable *in situ* tumors into clinical cancer disease. It has long been known that exposure to sex steroids increase the risk of breast cancer while early oophorectomy may reduce the risk of this disease by up to 60% [39], [40]. Ovarian ablation is, however, associated with osteoporosis and cardiovascular disease as well as vasomotor symptoms, urogenital atrophy, and decrease quality of life. Tamoxifen has been shown to reduce the incidence of breast cancer by more than 40% but tamoxifen therapy may induce severe side effects such as endometrial cancer and thromboembolism [20], [41]. Clearly there is a need to develop safer and less toxic breast cancer preventive strategies.

In our present data tamoxifen had significant effects on the pro-angiogenic proteins investigated here. Both angiogenin and VEGF levels were 22–25% lower in breast tissue after tamoxifen therapy for six weeks. Overall there were very low levels of VEGF in breast tissue, 5–80 pg/ml compared with angiogenin levels in the range of 3490–10300 pg/ml, and endostatin concentrations varying between 5721–15480 pg/ml in unexposed breast tissue. Although the *in vitro* recovery was approximately four times lower for VEGF this do not explain the differences in the tissue, hence the absolute levels for VEGF are much lower than those of the other proteins. As VEGF and angiogenin induce *in vivo* blood vessel growth at similar concentrations [9], [42] the higher angiogenin concentrations may be a physiologically more important pro-angiogenic factor *in situ* and a clinically more important factor to target.

Tamoxifen did also increase the endostatin levels by 33% in breast tissue. Clinical trials using recombinant endostatin for cancer therapy have failed for patients in an advanced stage [43]. Although endostatin yet has to be proven efficient for cancer therapy the significance of this protein may be in prevention of cancer. Individuals with endogenous higher endostatin levels due to increased copy numbers of the gene coding for collagen XVIII have reduced risk of solid tumors [44] and endostatin deficient mice exhibit decreased tumor growth rate compared with wild-type mice [45].

Diet modifications may be another route for breast cancer prevention associated with less side-effects compared with drugs such as tamoxifen. We have recently shown that a diet of 10% flaxseed to mice bearing estrogen dependent breast cancer explants reduces growth and angiogenesis by affecting angiogenesis regulators including VEGF [46], [47], [48]. Moreover, a daily addition of 25 g of flaxseed for 30 days to postmenopausal women newly diagnosed with breast cancer has been shown to attenuate several biological tumor markers [49].

In this study we wanted to investigate if anti-angiogenic effects similar to that of tamoxifen could be induced by dietary flaxseed in healthy volunteers. Our present data did, however, not show any effects of VEGF levels in breast tissue after the addition of flaxseed to the diet. The difference compared with the animal data, where VEGF levels decreased by flaxseed, may be a difference of cancerous tissue versus normal tissue and the fact that there is a magnitude of difference in lignan exposure in women (∼1 mg/kg bwt) and mice (∼10 mg/kg bwt). Moreover, in animal studies, the amount of flaxseed added to the diet equates approximately 10% of the daily caloric intake while in women 25 g of ground flaxseed is less than 5% of the total calorie intake. However, in humans, continuous flaxseed ingestion above 30 g/day is not recommended due to the presence of endogeneous cyanogenic compounds in seeds and the plant's disposition to accumulate cadmium from soil. The total lignan content in the flaxseed used in our study was equal to levels previously reported [50]. There are several metabolically active components in flaxseed and it has been reported that flaxseed may affect estrogen metabolism in postmenopausal women [51]. In our study we were not able to detect any differences in estradiol and/or progesterone levels after flaxseed ingestion in the women ruling out an effect of flaxseed on estrogen levels in our data. The levels of enterodiol and enterolactone after flaxseed ingestion confirmed the compliance with the diet. However, there were no significant correlation between serum levels of secoisolariciresinol, enterodiol, enterolactone or the total enterolignan levels and endostatin in breast tissue. This may suggest that there is no strong dose-response relationship with endostatin and lignans, or the cohort is to small to detect a possible relationship or, that other component(s) in flaxseed, not measured in our study, may modulate endostatin in breast tissue.

In summary, this study shows for the first time that extracellular levels of endostatin in normal human breast tissue *in vivo* exhibited a significant negative correlation with estradiol. Tamoxifen decreased the pro-angiogenic factors angiogenin and VEGF, and increased the levels of the anti-angiogenic endostatin in normal breast tissue. An addition of 25 grams of ground flaxseed to healthy volunteers during one menstrual cycle did not affect the levels of angiogenin and VEGF in normal breast tissue but increased the levels of endostatin in a similar fashion as tamoxifen. These results reveal previously unknown mechanisms of tamoxifen in the normal breast. A diet modification with flaxseed for one menstrual cycle does not seem to be equally powerful as tamoxifen in tipping the angiogenic balance towards angiogenesis inhibition in normal breast tissue. Further studies of diet alterations, perhaps of a longer duration, for elucidating effects on normal breast tissue for breast cancer prevention are warranted.

## References

[1] Collaborative Group on Hormonal Factors in Breast Cancer (1997). Breast cancer and hormone replacement therapy: collaborative reanalysis of data from 51 epidemiological studies of 52,705 women with breast cancer and 108,411 women without breast cancer.. Lancet.

[2] Rossouw JE, Anderson GL, Prentice RL, LaCroix AZ, Kooperberg C (2002). Risks and benefits of estrogen plus progestin in healthy postmenopausal women: principal results From the Women's Health Initiative randomized controlled trial.. Jama.

[3] Folkman J (1992). The role of angiogenesis in tumor growth.. Semin Cancer Biol.

[4] Dabrosin C (2003). Variability of Vascular Endothelial Growth Factor in Normal Human Breast Tissue in Vivo during the Menstrual Cycle.. J Clin Endocrinol Metab.

[5] Dabrosin C (2005). Positive correlation between estradiol and vascular endothelial growth factor but not fibroblast growth factor-2 in normal human breast tissue in vivo.. Clin Cancer Res.

[6] Hyder SM, Nawaz Z, Chiappetta C, Stancel GM (2000). Identification of functional estrogen response elements in the gene coding for the potent angiogenic factor vascular endothelial growth factor.. Cancer Res.

[7] Dabrosin C, Margetts PJ, Gauldie J (2003). Estradiol increases extracellular levels of vascular endothelial growth factor in vivo in murine mammary cancer.. Int J Cancer.

[8] Dabrosin C, Palmer K, Muller WJ, Gauldie J (2003). Estradiol promotes growth and angiogenesis in polyoma middle T transgenic mouse mammary tumor explants.. Breast Cancer Res Treat.

[9] Fett JW, Strydom DJ, Lobb RR, Alderman EM, Bethune JL (1985). Isolation and characterization of angiogenin, an angiogenic protein from human carcinoma cells.. Biochemistry.

[10] Kishimoto K, Liu S, Tsuji T, Olson KA, Hu GF (2005). Endogenous angiogenin in endothelial cells is a general requirement for cell proliferation and angiogenesis.. Oncogene.

[11] Xu ZP, Tsuji T, Riordan JF, Hu GF (2002). The nuclear function of angiogenin in endothelial cells is related to rRNA production.. Biochem Biophys Res Commun.

[12] Nilsson UW, Abrahamsson A, Dabrosin C (2010). Angiogenin regulation by estradiol in breast tissue: tamoxifen inhibits angiogenin nuclear translocation and antiangiogenin therapy reduces breast cancer growth in vivo.. Clin Cancer Res.

[13] O'Reilly MS, Boehm T, Shing Y, Fukai N, Vasios G (1997). Endostatin: an endogenous inhibitor of angiogenesis and tumor growth.. Cell.

[14] Sasaki T, Larsson H, Tisi D, Claesson-Welsh L, Hohenester E (2000). Endostatins derived from collagens XV and XVIII differ in structural and binding properties, tissue distribution and anti-angiogenic activity.. J Mol Biol.

[15] Dhanabal M, Ramchandran R, Waterman MJ, Lu H, Knebelmann B (1999). Endostatin induces endothelial cell apoptosis.. J Biol Chem.

[16] Hajitou A, Grignet C, Devy L, Berndt S, Blacher S (2002). The antitumoral effect of endostatin and angiostatin is associated with a down-regulation of vascular endothelial growth factor expression in tumor cells.. Faseb J.

[17] Nilsson UW, Dabrosin C (2006). Estradiol and tamoxifen regulate endostatin generation via matrix metalloproteinase activity in breast cancer in vivo.. Cancer Res.

[18] Garvin S, Nilsson UW, Huss FR, Kratz G, Dabrosin C (2006). Estradiol increases VEGF in human breast studied by whole-tissue culture.. Cell Tissue Res.

[19] Bendrik C, Robertson J, Gauldie J, Dabrosin C (2008). Gene transfer of matrix metalloproteinase-9 induces tumor regression of breast cancer in vivo.. Cancer Res.

[20] Fisher B, Costantino JP, Wickerham DL, Redmond CK, Kavanah M (1998). Tamoxifen for prevention of breast cancer: report of the National Surgical Adjuvant Breast and Bowel Project P-1 Study.. J Natl Cancer Inst.

[21] Cuzick J, Forbes J, Edwards R, Baum M, Cawthorn S (2002). First results from the International Breast Cancer Intervention Study (IBIS-I): a randomised prevention trial.. Lancet.

[22] Cuzick J, Powles T, Veronesi U, Forbes J, Edwards R (2003). Overview of the main outcomes in breast-cancer prevention trials.. Lancet.

[23] Althuis MD, Dozier JM, Anderson WF, Devesa SS, Brinton LA (2005). Global trends in breast cancer incidence and mortality 1973-1997.. Int J Epidemiol.

[24] Howe GR, Hirohata T, Hislop TG, Iscovich JM, Yuan JM (1990). Dietary factors and risk of breast cancer: combined analysis of 12 case- control studies.. J Natl Cancer Inst.

[25] Saarinen NM, Tuominen J, Santti R, Pylkkänen L, Yagasaki K, Yamazaki M (2008). Anticarcinogenic effects of lignans in breast and prostate – a critical review of the current knowledge.. Kerala, India: Research Signpost.

[26] Dabrosin C (2001). Technical aspects of microdialysis of human breast.. Scand J Clin Lab Invest.

[27] Dabrosin C, Hallstrom A, Ungerstedt U, Hammar M (1997). Microdialysis of human breast tissue during the menstrual cycle.. Clin Sci (Lond).

[28] Dabrosin C, Ollinger K, Ungerstedt U, Hammar M (1997). Variability of glutathione levels in normal breast tissue and subcutaneous fat during the menstrual cycle: an in vivo study with microdialysis technique.. J Clin Endocrinol Metab.

[29] Rosdahl H, Hamrin K, Ungerstedt U, Henriksson J (1998). Metabolite levels in human skeletal muscle and adipose tissue studied with microdialysis at low perfusion flow.. Am J Physiol.

[30] Nurmi T, Adlercreutz H (1999). Sensitive high-performance liquid chromatographic method for profiling phytoestrogens using coulometric electrode array detection: application to plasma analysis.. Anal Biochem.

[31] Penalvo JL, Nurmi T, Haajanen K, Al-Maharik N, Botting N (2004). Determination of lignans in human plasma by liquid chromatography with coulometric electrode array detection.. Anal Biochem.

[32] Nurmi T, Voutilainen S, Nyyssonen K, Adlercreutz H, Salonen JT (2003). Liquid chromatography method for plant and mammalian lignans in human urine.. J Chromatogr B Analyt Technol Biomed Life Sci.

[33] Penttinen-Damdimopoulou PE, Power KA, Hurmerinta TT, Nurmi T, van der Saag PT (2009). Dietary sources of lignans and isoflavones modulate responses to estradiol in estrogen reporter mice.. Mol Nutr Food Res.

[34] Dabrosin C (2003). Increase of free insulin-like growth factor-1 in normal human breast in vivo late in the menstrual cycle.. Breast Cancer Res Treat.

[35] Dabrosin C (2005). Increased extracellular local levels of estradiol in normal breast in vivo during the luteal phase of the menstrual cycle.. J Endocrinol.

[36] Dabrosin C (2005). Microdialysis - an in vivo technique for studies of growth factors in breast cancer.. Front Biosci.

[37] Garvin S, Dabrosin C (2003). Tamoxifen inhibits secretion of vascular endothelial growth factor in breast cancer in vivo.. Cancer Res.

[38] Welch HG, Black WC (1997). Using autopsy series to estimate the disease “reservoir” for ductal carcinoma in situ of the breast: how much more breast cancer can we find?. Ann Intern Med.

[39] Hulka BS, Stark AT (1995). Breast cancer: cause and prevention.. Lancet.

[40] Pike MC, Pearce CL, Wu AH (2004). Prevention of cancers of the breast, endometrium and ovary.. Oncogene.

[41] (1998). Tamoxifen for early breast cancer: an overview of the randomised trials. Early Breast Cancer Trialists' Collaborative Group.. Lancet.

[42] Connolly DT, Heuvelman DM, Nelson R, Olander JV, Eppley BL (1989). Tumor vascular permeability factor stimulates endothelial cell growth and angiogenesis.. J Clin Invest.

[43] Kulke MH, Bergsland EK, Ryan DP, Enzinger PC, Lynch TJ (2006). Phase II study of recombinant human endostatin in patients with advanced neuroendocrine tumors.. J Clin Oncol.

[44] Zorick TS, Mustacchi Z, Bando SY, Zatz M, Moreira-Filho CA (2001). High serum endostatin levels in Down syndrome: implications for improved treatment and prevention of solid tumours.. Eur J Hum Genet.

[45] Sund M, Hamano Y, Sugimoto H, Sudhakar A, Soubasakos M (2005). Function of endogenous inhibitors of angiogenesis as endothelium-specific tumor suppressors.. Proc Natl Acad Sci U S A.

[46] Lindahl G, Saarinen N, Abrahamsson A, Dabrosin C (2011). Tamoxifen, flaxseed, and the lignan enterolactone increase stroma- and cancer cell-derived IL-1Ra and decrease tumor angiogenesis in estrogen-dependent breast cancer.. Cancer Res.

[47] Saarinen NM, Abrahamsson A, Dabrosin C (2010). Estrogen-induced angiogenic factors derived from stromal and cancer cells are differently regulated by enterolactone and genistein in human breast cancer in vivo.. Int J Cancer.

[48] Bergman Jungestrom M, Thompson LU, Dabrosin C (2007). Flaxseed and its lignans inhibit estradiol-induced growth, angiogenesis, and secretion of vascular endothelial growth factor in human breast cancer xenografts in vivo.. Clin Cancer Res.

[49] Thompson LU, Chen JM, Li T, Strasser-Weippl K, Goss PE (2005). Dietary flaxseed alters tumor biological markers in postmenopausal breast cancer.. Clin Cancer Res.

[50] Smeds AI, Eklund PC, Sjoholm RE, Willfor SM, Nishibe S (2007). Quantification of a broad spectrum of lignans in cereals, oilseeds, and nuts.. J Agric Food Chem.

[51] Brooks JD, Ward WE, Lewis JE, Hilditch J, Nickell L (2004). Supplementation with flaxseed alters estrogen metabolism in postmenopausal women to a greater extent than does supplementation with an equal amount of soy.. Am J Clin Nutr.

